# Design of a bacterial speck resistant tomato by CRISPR/Cas9‐mediated editing of *SlJAZ2*

**DOI:** 10.1111/pbi.13006

**Published:** 2018-10-05

**Authors:** Andrés Ortigosa, Selena Gimenez‐Ibanez, Nathalie Leonhardt, Roberto Solano

**Affiliations:** ^1^ Departamento de Genética Molecular de Plantas Centro Nacional de Biotecnología Consejo Superior de Investigaciones Científicas (CNB‐CSIC) Madrid Spain; ^2^ Unité Mixte de Recherche 7265 Laboratoire de Biologie du Développement des Plantes Commissariat à l'Energie Atomique et aux Energies alternatives (CEA) Cadarache CNRS/CEA/Aix‐Marseille Université Saint‐Paul‐lez‐Durance France

**Keywords:** coronatine, CRISPR/Cas9, JAZ2, pseudomonas, stomata, tomato

## Abstract

Due to their different lifestyles, effective defence against biotrophic pathogens normally leads to increased susceptibility to necrotrophs, and vice versa. Solving this trade‐off is a major challenge for obtaining broad‐spectrum resistance in crops and requires uncoupling the antagonism between the jasmonate (JA) and salicylate (SA) defence pathways. *Pseudomonas syringae* pv. *tomato* (*Pto*) DC3000, the causal agent of tomato bacterial speck disease, produces coronatine (COR) that stimulates stomata opening and facilitates bacterial leaf colonization. In *Arabidopsis,* stomata response to COR requires the COR co‐receptor AtJAZ2, and dominant AtJAZ2Δjas repressors resistant to proteasomal degradation prevent stomatal opening by COR. Here, we report the generation of a tomato variety resistant to the bacterial speck disease caused by *Pto* DC3000 without compromising resistance to necrotrophs. We identified the functional ortholog of AtJAZ2 in tomato, found that preferentially accumulates in stomata and proved that SlJAZ2 is a major co‐receptor of COR in stomatal guard cells. *SlJAZ2* was edited using CRISPR/Cas9 to generate dominant JAZ2 repressors lacking the C‐terminal Jas domain (SlJAZ2Δjas). SlJAZ2Δjas prevented stomatal reopening by COR and provided resistance to *Pto* DC3000. Water transpiration rate and resistance to the necrotrophic fungal pathogen *Botrytis cinerea*, causal agent of the tomato gray mold, remained unaltered in *Sljaz2Δjas* plants. Our results solve the defence trade‐off in a crop, by spatially uncoupling the SA‐JA hormonal antagonism at the stomata, entry gates of specific microbes such as *Pto* DC3000. Moreover, our results also constitute a novel CRISPR/Cas‐based strategy for crop protection that could be readily implemented in the field.

## Introduction

*Pseudomonas syringae* is a widespread bacterial pathogen that causes disease on a broad range of economically important plant species. Among them, *P. syringae* pv. *tomato* DC3000 (*Pto* DC3000) is the causative agent of the bacterial speck disease of tomato (*Solanum lycopersicum*) (Blancard, 2012). Susceptible tomato cultivars include the variety Moneymaker, which is agronomically and economically important. Outbreaks of bacterial speck on tomatoes occur in moderate temperatures (15–25°) and wet conditions (Jones *et al*., 1991). Under favourable environmental conditions, disease symptoms appear as small brown necrotic spots (specks) in leaf and fruits (Bender *et al*., 1987). This disease affects negatively the productivity and marketability of the tomatoes (Jones *et al*.,^1991^) and causes economic losses all over the world (Gitaitis *et al*., 1985; Schneider, 1977).

The initial infective process of *P. syringae* relies on natural openings and accidental wounds on the plant surface to colonize internal tissues (Melotto *et al*., 2008). Stomata are an example of such openings, providing one of the main routes through which the foliar pathogen *P. syringae* can penetrate the leaf epidermis and start multiplying aggressively in the apoplast (Melotto *et al*., 2017). Stomata are pores present on the surface of the leaves that are involved in gas exchange, regulating photosynthesis and water loss through transpiration stream (Kim *et al*., 2010). Besides this, stomata also play an active and dynamic role in defence against pathogens being an integral part of the plant innate immunity system (Liu *et al*., 2009; Melotto *et al*., 2006, 2008; Zhang *et al*., 2008). Upon microbial perception, achieved by the specific recognition of conserved microbial associated molecular patterns (MAMPS) such as bacterial flagellin, via surface‐localized receptors, plants rapidly close stomata (Melotto *et al*., 2006). This closure requires the salicylic acid (SA) and abscisic acid (ABA) plant hormones, and inhibits the entry of *P. syringae* restricting host tissue colonization (Du *et al*., 2014; Gimenez‐Ibanez *et al*., 2017; Melotto *et al*., 2006; Zeng and He, 2010; Zhang *et al*., 2008). Once in the apoplast, *P. syringae* encounters apoplastic plant immunity, which also relies on a plethora of plant hormones including SA and jasmonic acid (JA). In general terms, SA defences positively regulate resistance to biotrophic and hemi‐biotrophic microbes such as *P. syringae*, whereas a combination of JA and ethylene (ET) pathways activates resistance against necrotrophic pathogens such as the fungus *Botrytis cinerea* (Robert‐Seilaniantz *et al*., 2011). SA and JA/ET defence pathways generally antagonize each other, and consequently, elevated resistance to biotrophs is often correlated with increased susceptibility to necrotrophs, and vice versa (Glazebrook, 2005).

*Pseudomonas syringae* strains such as *Pto* DC3000 have evolved a refined strategy for manipulating hormonal crosstalk by producing coronatine (COR), a mimic of the bioactive JA hormone, JA‐isoleucine (JA‐Ile) (Fonseca *et al*., 2009a). By activating the JA pathway, COR inhibits SA‐dependent defences and thus stimulates the reopening of stomata to facilitate bacterial invasion and growth in the apoplast (Brooks *et al*., 2005; Gimenez‐Ibanez *et al*., 2017; Laurie‐Berry *et al*., 2006; Melotto *et al*., 2006, 2008; Zheng *et al*., 2012). The signalling cascade triggered by COR and JA‐Ile is well established. COR is perceived by a co‐receptor formed by the F‐box protein COI1 (CORONATINE‐INSENSITIVE 1) and JAZ (JASMONATE ZIM DOMAIN) repressor proteins (Chini *et al*., 2007; Katsir *et al*., 2008; Sheard *et al*., 2010; Thines *et al*., 2007; Xie *et al*., 1998). JAZ co‐receptors are COI1 substrates that negatively regulate the JA‐signalling pathway by directly interacting with and repressing transcription factors (TFs) such as MYC2/3/4 that control JA‐regulated genes (Chini *et al*., 2007; Fernández‐Calvo *et al*., 2011; Lorenzo *et al*., 2004; Sheard *et al*., 2010; Thines *et al*., 2007). Repression of TFs by JAZ is mediated by recruitment of the TOPLESS (TPL) co‐repressor complex, through the adaptor protein NINJA. JAZ also prevent the interaction of the TFs with the MEDIATOR complex through MED25 (Cevik *et al*., 2012; Chen *et al*., 2012; Pauwels *et al*., 2010; Zhang *et al*., 2015). Under stress conditions, COR or JA‐Ile promote the formation of JAZ‐COI1 complexes, triggering JAZ degradation via the 26S proteasome (Chini *et al*., 2007; Sheard *et al*., 2010; Thines *et al*., 2007). This leads to de‐repression of the TFs that initiate the transcription of JA‐dependent genes, and repression of SA‐dependent defences against the bacteria (Fonseca *et al*., 2009b; Gimenez‐Ibanez and Solano, 2013). Thus, COR acts as a potent virulence factor in plants by triggering the degradation of JAZs. Acquisition of COR by bacterial pathogens has been of tremendous adaptive importance during host‐pathogen evolution because it has allowed bacteria to manipulate the host hormonal network to promote susceptibility.

Among JAZ repressors, we recently showed that AtJAZ2 is a major COR/JA‐Ile co‐receptor in Arabidopsis controlling stomata dynamics during bacterial invasion. JAZ2 is constitutively expressed at the stomata and hijacked by bacterially produced COR to suppress SA‐dependent stomatal closure and promote bacterial penetration (Gimenez‐Ibanez *et al*., 2017). Arabidopsis *jaz2* loss‐of‐function mutants are partially impaired in pathogen‐induced stomatal closing and more susceptible to *Pseudomonas*. In contrast, truncated JAZ2 forms lacking the C‐terminal Jas domain (JAZ2Δjas) act as gain‐of‐function mutations that prevent stomatal reopening by COR and are highly resistant to bacterial penetration. The C‐terminal Jas domain is responsible for the interaction with COI1 in the presence of hormone and thus, JAZ forms lacking this conserved domain are resistant to COR‐induced degradation, overcoming the action of the phytotoxin during the entry process of *P. syringae* (Gimenez‐Ibanez *et al*., 2017). The fact that JAZ2 function is mostly restricted to the stomata makes that alterations in this gene do not affect the SA‐JA crosstalk in other tissues and, therefore, *jaz2Δjas* mutants still retain unaltered resistance against necrotrophs. Our work suggested that gain‐of‐function mutations in JAZ2 could be used as a general strategy to spatially uncouple SA‐JA hormonal antagonism and to block the entry of *P. syringae* strains that produce COR without compromising resistance to necrotrophs, which is mostly apoplastic.

Genome editing technologies such as CRISPR/Cas9 (Clustered Regularly Interspaced Short Palindromic Repeats/CRISPR‐associated endonuclease 9) enable precise and directed modifications of DNA sequences in vivo (Schiml and Puchta, 2016). CRISPR/Cas is emerging as an accurate method to improve crops because of its specificity, simplicity and versatility. The Cas9 protein functions as a nuclease and is directed to a target site by a specific guide RNA (gRNA), inducing site‐specific double‐strand breaks. This damage can be then repaired by non‐homologous end‐joining (NHEJ) or homologous recombination (HR), but in the case of NHEJ, the process of repair is error prone, which results in disruptive insertions or deletions at targeted loci that can result in translational frame shifts, amino acid replacements or deletions (Mahfouz *et al*., 2014).

Genetic manipulation of defence pathways has succeeded in enhancing resistance to specific kinds of pathogens. However, due to the antagonistic interactions between the SA and JA defence pathways, efforts to develop plants with broad‐spectrum resistance by manipulation of hormonal signalling genes has had limited success so far because enhancement of the SA‐dependent defences leads to a reduction in JA‐based resistance and vice versa (Robert‐Seilaniantz *et al*., 2011; Pieterse *et al*., 2012). Solving this trade‐off is a major challenge in agriculture and requires uncoupling the antagonism between hormonal pathways in crops.

In this study, we report on a solution to this trade‐off by spatially uncoupling the SA‐JA antagonism at the stomata and generating a tomato (Moneymaker) resistant to the bacterial speck disease caused by the pathogen *Pto* DC3000, without compromising resistance to necrotrophic pathogens. We identified the functional ortholog of the COR stomatal co‐receptor AtJAZ2 in tomato (*SlJAZ2*) and edited this gene with the CRISPR/Cas9 system to generate truncated JAZ2 forms lacking the C‐terminal Jas domain (SlJAZ2Δjas). This edited gain‐of‐function SlJAZ2Δjas mutant fully prevented stomatal reopening by COR and reduced bacterial entry through the stomata, increasing resistance to *Pto* DC3000 infection. Stomatal aperture in *Sljaz2*‐edited plants remained unaltered during transpiration. Moreover, since our strategy does not affect JA‐signalling outside the stomata, *Sljaz2Δjas* plants showed unaltered levels of resistance to the necrotrophic fungal pathogen *B. cinerea*, causal agent of the tomato gray mold. In addition to uncouple the SA‐JA defence antagonism in a crop, our results constitute a novel strategy for crop protection using CRISPR/Cas that could be readily implemented in the field.

## Results

### SlJAZ2 is the ortholog of AtJAZ2

**Figure 1:**
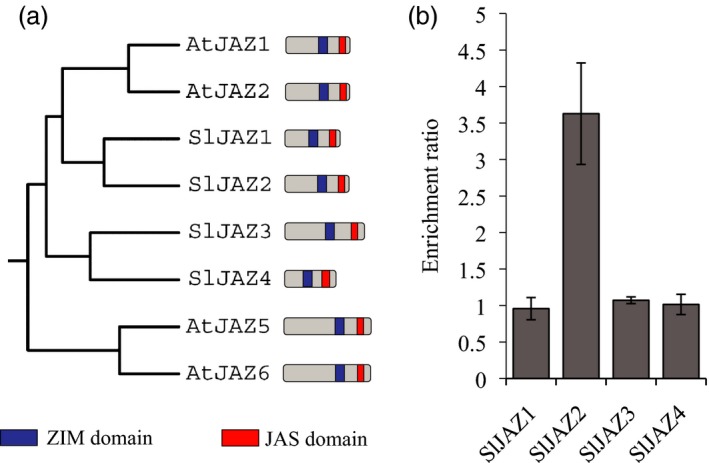
SlJAZ2 is the ortholog of AtJAZ2. (a) Phylogenetic tree of JAZ1, JAZ2, JAZ3 and JAZ4 from Arabidopsis and tomato. SlJAZ1 and SlJAZ2 are the closest proteins to AtJAZ2. The schematic representation indicates length and domains of each JAZ protein. (b) Enrichment ratio of RT‐PCR analyses comparing expression levels of *SlJAZ1*,* SlJAZ2*,* SlJAZ3* and *SlJAZ4* in tomato whole leaf tissue and epidermal peels, which are enriched in guard cells. This experiment was repeated twice with similar results.

The AtJAZ2 protein belongs to the TIFY super‐family that includes 12 canonical members in Arabidopsis. Similarly, 12 JAZ proteins have also been identified in tomato (Chini *et al*., 2017; Ishiga *et al*., 2013; Sun *et al*., 2011). However, correlation between specific versions of *JAZ* genes among plant species is unclear. In order to identify the ortholog of AtJAZ2 in tomato we performed phylogenetic analysis using protein sequences of all JAZs from both plant species. AtJAZ2 protein grouped into a clade that included AtJAZ1, and the SlJAZ1 and SlJAZ2 proteins as the closest tomato orthologs (Figure 1a and Figure S1). SlJAZ3 and SlJAZ4 were also closer to AtJAZ2 than to any other tomato or Arabidopsis protein. Among proteins in this clade, AtJAZ1, AtJAZ2 and SlJAZ2 showed a remarkable similar length (Figure 1a). This phylogenetic analysis indicated that the ortholog of AtJAZ2 in tomato was likely SlJAZ2 or SlJAZ1, although SlJAZ3 and SlJAZ4 cannot be discarded.

*JAZ2* is strongly expressed at stomatal guard cells compared to other *JAZ*s in Arabidopsis (Gimenez‐Ibanez *et al*., 2017). Thus, we next analysed if any of the closest tomato candidate orthologs of *AtJAZ2* were expressed at the stomata. To do this, we performed quantitative RT‐PCR analyses that compared the expression levels of these genes between tomato whole leaf tissue and epidermal peels, which are enriched in guard cells. Sl*JAZ2* was the only gene enriched in the stomata‐abundant epidermal peels fraction whereas Sl*JAZ1*, Sl*JAZ3* and Sl*JAZ4* relative levels were similar among them (Figure 1b). This pinpointed *SlJAZ2* as the functional tomato ortholog of AtJAZ2.

### *SLJAZ2* editing through CRISPR/Cas9

**Figure 2:**
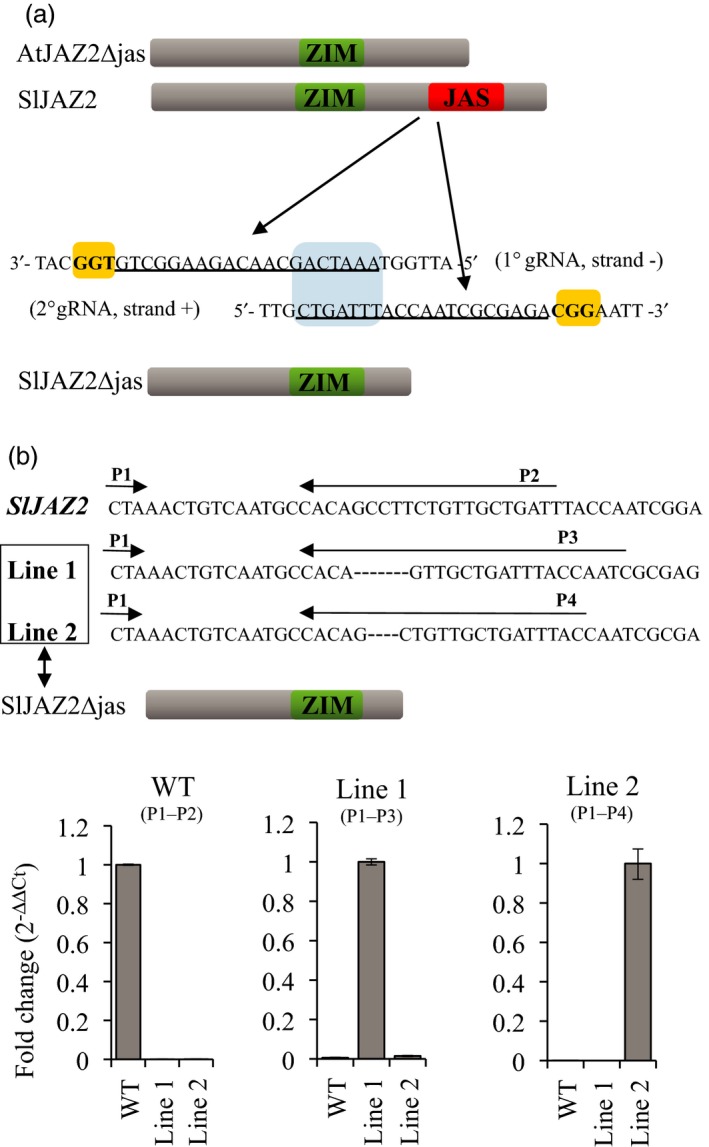
CRISPR/Cas9‐mediated editing of *SlJAZ2*. (a) Schematic representation of AtJAZ2Δjas, SlJAZ2, SlJAZ2Δjas, and position of both gRNAs designed to target the Jas domain in the *SlJAZ2* locus. Nucleotide sequence indicates double strand DNA of *SlJAZ2*. Yellow boxes indicate the PAM sequence. Underlined sequence indicates the two gRNAs used. The 1° gRNA is targeted before the start of the Jas domain (strand −) while the 2°gRNA is targeted to the beginning of the Jas domain (strand +). The target area of both gRNAs overlaps seven nucleotides (blue box). (b) Transcriptional expression by RT‐PCR of SlJAZ2 and *SlJAZ2Δjas* forms in WT and *SlJaz2Δjas* plants (Line 1 and Line 2) using different combinations of primers as described in the figure.

In Arabidopsis, a truncated form of AtJAZ2 lacking the Jas domain (AtJAZ2ΔJas) is resistant to AtCOI1‐dependent degradation after COR treatment. Arabidopsis plants overexpressing this degradation resistant JAZ2 dominant form are insensitive to the phytotoxin COR and more resistant to DC3000 (Gimenez‐Ibanez *et al*., 2017). We used CRISPR/Cas9 genome‐editing technology to generate sequence‐specific mutations that would disrupt the Jas domain of SlJAZ2, leading to a *JAZ2ΔJas* tomato variant in the commercial variety Moneymaker. To do this, we targeted the start site of the Jas domain in the *SlJAZ2* locus using a dual gRNA strategy that facilitates the generation of homozygous deletions (Brooks *et al*., 2014). The two selected gRNA targets within SlJAZ2 were positioned on opposite DNA strands, and overlapped 7 nucleotides (Figure 2a). DNA sequence analysis of primary transformants identified two homozygous lines for *SlJAZ2* containing deletions of seven and four nucleotides in the designated area (Figure 2b). Both deletions were predicted to disrupt the Jas domain of *SlJAZ2* (Figure 2b). We further explored if these genome edited *Sljaz2Δjas* mutations were specifically being expressed in our tomato lines. To do this, we performed quantitative RT‐PCR experiments amplifying wild‐type (WT) *SlJAZ2* or each one of the two specific *Sljaz2Δjas* mutations generated in both tomato lines. WT, Line 1 and Line 2 amplified exclusively their expected transcripts (Figure 2b), indicating both lines carry homozygous mutations at the *SlJAZ2* locus generating forms of *SlJAZ2Δjas* that are effectively being transcribed. Further analysis of T1 and T2 *Sljaz2Δjas* lines by DNA sequencing of *SlJAZ2* showed that the homozygous mutations were stably transmitted to the offspring.

### Gain‐of‐function *Sljaz2Δjas* prevent stomatal reopening by COR

**Figure 3:**
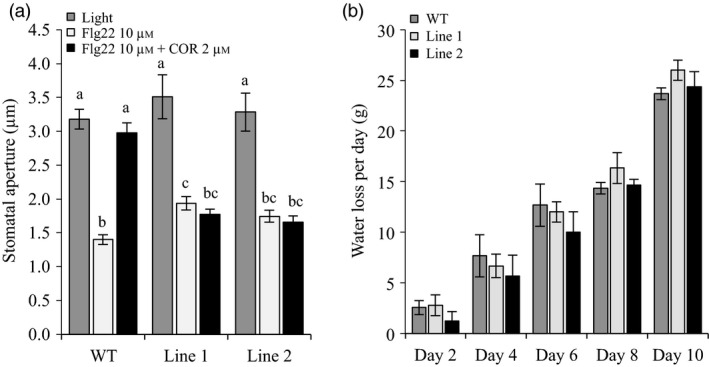
*Sljaz2Δjas* mutants prevent stomatal reopening by COR. (a) Stomatal aperture in tomato WT and *Sljaz2Δjas* mutants (Line 1 and Line 2) after 2 h of incubation with flg22, flg22 plus COR or a mock control. Error bars indicate standard error of the mean (SEM;* n* = 60). Letters above bars represent statistically distinct groups (*P* < 0.01, one‐way Anova with Tukey‐HSD). Similar results were obtained in three independent biological replicates (b) Water transpiration rate (water loss) in WT and *Sljaz2Δjas* (Line 1 and Line 2) tomato plants during a time course experiment of 10 days. Error bars indicate standard deviation (SD;* n* = 3). Not statistical difference was found between WT and mutant lines (one‐way Anova with Tukey‐HSD). Results are representative of two independent experiments.

Pathogen perception induces stomatal closure to limit pathogen penetration. Some *P. syringae* strains produce COR as a critical virulence strategy to re‐open stomata and cause disease. Thus, we next evaluated the ability of both *Sljaz2Δjas* tomato lines to close stomata upon perception of the highly conserved flg22 peptide of bacterial flagellum (Chinchilla *et al*., 2007), and to reopen them in the presence of COR. We incubated leaves of WT plants and *Sljaz2Δjas* mutants with flg22, flg22 plus COR or a mock solution as a control. In mock conditions, the aperture range of the stomata was wide and similar between WT and both *Sljaz2Δjas* tomato lines. As expected, flg22 induced stomatal closure in all tomatoes, whereas COR induced stomatal re‐opening in WT plants (Figure 3a). In contrast to WT, both *Sljaz2Δjas* tomato lines were fully impaired in COR‐mediated stomatal reopening (Figure 3a). This indicates that, similar to Arabidopsis, SlJAZ2 has a key role regulating stomata dynamics in response to bacteria and that COR‐induced stomata reopening requires inhibition of *SlJAZ2,* which cannot be achieved in the dominant *Sljaz2Δjas* mutants.

Stomata are the major regulators of water transpiration in plants (Brodribb and McAdam, 2017). We next evaluated whether the rate of transpiration was affected in *Sljaz2Δjas* lines compared to WT tomato plants. Time‐course measurements showed that the rate of water loss in all plants was similar (Figure 3b). We also analysed leaf temperature, which is an indirect measure of stomata opening and water transpiration, using infrared thermography and found no differences related to stomatal apertures (Figure S2). Overall, this indicates that *Sljaz2Δjas* tomato lines are not affected in the aperture of the stomata during the process of transpiration, but are rather specifically affected in the response to bacteria.

### Dominant *Sljaz2Δjas* mutants are resistant to *P. syringae* infection

**Figure 4:**
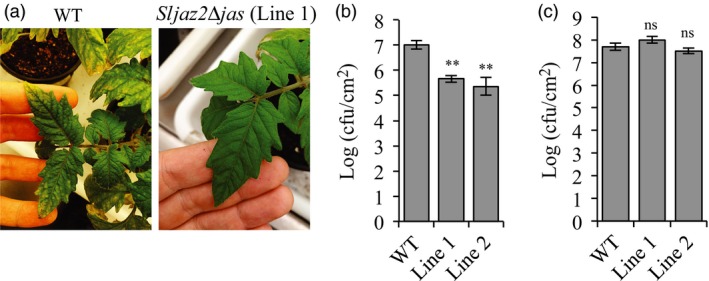
*Sljaz2Δjas* mutants are more resistant to *Pto* DC3000 when surface inoculated. (a) *Pto* DC3000 disease symptoms on WT and *Sljaz2Δjas* (Line 1) tomato plants after surface inoculation with *Pto* DC3000 bacteria at 10^8^ colony‐forming units/mL (cfu/mL). Pictures were taken 6 days postinoculation and show typical specks caused by *Pto* DC3000. (b) Growth of *Pto* DC3000 on WT and *Sljaz2Δjas* (Line 1 and Line 2) tomato plants 6 days after surface inoculation by dipping with bacteria at 10^8^ cfu/mL. (c) Growth of *Pto* DC3000 on WT and *Sljaz2Δjas* (Line 1 and Line 2) tomato plants 2 days after syringe infiltration with bacteria at 5 × 10^5^ cfu/mL. Error bars indicate SEM (*n* = 7). Asterisks represent significant differences **P < 0.01 (Student's *t*‐test). Similar results were obtained in three independent experiments and representative results are shown.

In order to evaluate the resistance of adult *Sljaz2Δjas* plants towards bacterial pathogens such as *P. syringae*, we next compared bacterial replication of *Pto* DC3000 on WT and both *Sljaz2Δjas* lines infected by surface inoculation (dipping) or syringe infiltration. Surface infection mimics natural infection conditions and it is a very common technique to assess plant resistance to pathogens (Zipfel *et al*., 2004). In contrast, the infiltration technique overcomes the early stomatal level of regulation measuring mainly apoplastic cell‐based defences. WT plants infected by surface inoculation with *Pto* DC3000 showed extensive chlorosis and specks, typical symptoms of the bacterial speck disease in tomatoes (Figure 4a). In contrast, *Sljaz2Δjas* plants did not display these typical symptoms of disease suggesting their enhanced resistance (Figure 4a). Disease symptoms correlated well with bacterial titres. *Sljaz2Δjas* plants surface inoculated showed significantly lower bacterial titres than those in its respective WT control plants (Figure 4b). In the case of plants infected with *Pto* DC3000 by infiltration, bacteria grew to similar levels on both WT and *Sljaz2Δjas* lines (Figure 4c). Consistently, inoculation with the coronatine‐deficient *Pto* DC3000 strain AK87 (*Pto* DC3000 *COR−*) showed similar bacterial titles in WT and *Sljaz2Δjas* plants (Figure S3). These results supports the idea that SlJAZ2 is a key regulator of stomata dynamics during the penetration process of *Pto* DC3000 and indicates that *Sljaz2Δjas* plants promote bacterial resistance by blocking the action of the phytotoxin COR at the stomata.

### Dominant *Sljaz2Δjas* tomato plants show unaltered levels of resistance against the necrotrophic pathogen *Botrytis cinerea*

**Figure 5:**
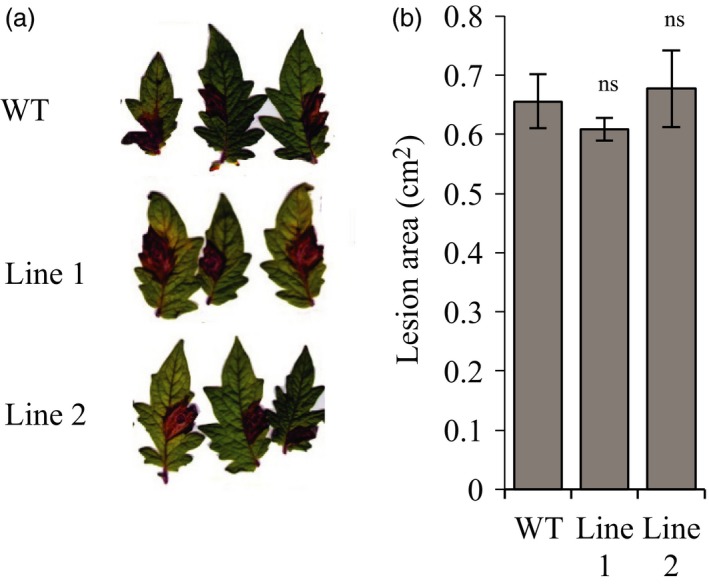
*Sljaz2Δjas* mutants retain WT resistance to necrotrophic fungi. (a) *Botrytis cinerea* disease symptoms on WT and *Sljaz2Δjas* mutants 4 days postinoculation (dpi) with 5 × 10^6^ spores m/L. (b) Lesion Area produced by *Botrytis cinerea* on WT and *Sljaz2Δjas* mutants 4 days postinoculation (dpi) with 5 × 10^6^ spores m/L. Error bars indicate standard error SEM (*n* = 8). (Student's *t*‐test, *P* < 0.05; ns, not significant). Results are representative of two independent experiments.

The antagonism between JA and SA pathways is a barrier to improve plant immunity and generate broad‐spectrum resistance. To evaluate if the insensitivity to COR generated in the edited *Sljaz2Δjas* tomato lines affects resistance against necrotrophic fungal pathogens, we selected *B. cinerea,* causal agent of the gray mold of tomatoes. Thus, we measured the susceptibility of WT and *Sljaz2Δjas* lines to *B. cinerea* by quantifying leaf infected area. Lesions caused by the fungus indicated similar progression of disease symptoms (Figure 5a) and area affected by the pathogen in WT and *Sljaz2Δjas* lines (Figure 5b). These results indicate that SlJAZ2 function seems restricted to stomata guard cells and it plays a very limited or inexistent role in apoplastic defence responses against necrotrophic fungi.

## Discussion

One of the great challenges for food security in the 21st century is to improve yield through the development of disease‐resistant crops. However, novel strategies to improve plant immunity are particularly challenging. In vascular plants, hormonal crosstalk between JA and SA, the key two hormonal pathways controlling resistant to vastly all type of pathogens, often antagonize each other, and thus, enhanced resistance against biotrophic pathogens normally leads to increased susceptibility to necrotrophs, and vice versa (Glazebrook, 2005). Solving this trade‐off is a major challenge for obtaining broad‐spectrum resistance and requires uncoupling the antagonism between these hormonal pathways in crops.

*Pto* DC3000 is the natural causal agent of the bacterial speck disease of tomato. Bacterial spots on tomato fruits have been reported to cause up to 52% loss of fruit weight (Jones *et al*., 1986), whereas in the field, yield losses due to bacterial speck vary from 75% in plants infected at an early stage of growth to 5% in plants infected later in the season (Yunis *et al*., 1980). Therefore, it becomes necessary to uncover novel sources of resistance against this pathogen. *Pto* DC3000 uses COR as a virulent factor to induce stomata opening and facilitate entrance into the leaf apoplast. We have recently identified the specific JAZ co‐receptor of COR at the stomata in Arabidopsis (Gimenez‐Ibanez *et al*., 2017). A dominant mutation of this stomata‐specific AtJAZ2 lacking a C‐terminal Jas regulatory domain (*Atjaz2Δjas*) is insensitive to COR and cannot re‐open stomata after bacterial infection. This mutation confers resistance to biotrophic *Pto* DC3000 without affecting susceptibility to necrotrophs, due to its specific action at guard cells (Gimenez‐Ibanez *et al*., 2017).

In this study, we demonstrate that similar *Δjas* mutations in stomata‐accumulating JAZ proteins can be used as a general strategy to increase resistance to COR‐producing bacteria in crops. Thus, as a proof‐of‐concept, we identified the tomato ortholog of *AtJAZ2* and successfully generated a bacterial speck resistant tomato in the commercial variety Moneymaker by CRISPR/Cas9‐mediated *SlJAZ2* editing. *Sljaz2Δjas* plants were significantly more resistant to *Pto* DC3000 when plants where surface inoculated, but equally susceptible by infiltration. This indicates that this SlJAZ2 variant is mainly affecting the stomatal defence layer by counteracting the effect of COR. Consistently, *Sljaz2Δjas* tomato lines were fully impaired in COR‐mediated stomatal reopening. Remarkably, since the *Sljaz2Δjas* mutation only manipulates the JA‐dependent defences at the stomata, the JA‐SA antagonism is not affected in the mesophyll and, therefore, apoplastic resistance to the necrotrophic pathogen *B. cinerea* remains unaffected. These results suggest that similar to Arabidopsis, SlJAZ2 plays a major role in regulating stomatal aperture during biotic stresses, and that COR produced by *Pto* DC3000 hijacks SlCOI1‐SlJAZ2 co‐receptor to promote the entry of the bacteria to the internal tissues of tomato leaf. Moreover, these results prove that is possible to uncouple SA‐JA antagonism spatially to design novel strategies for protection against COR‐producing *P. syringae* strains in crops, without affecting resistance to necrotrophs.

Alteration of the regulation of plant stomatal aperture can lead to deleterious effects. A number of environment factors, including CO_2_ level, light, water and other abiotic stresses also regulate stomatal dynamics. In all these cases, ABA functions as a chemical messenger that induces stomata closure (Lim *et al*., 2015; Vishwakarma *et al*., 2017). However, several lines of evidence suggest that *Sljaz2Δjas* tomato lines are not affected in these ABA‐mediated physiological processes. First, in Arabidopsis, gain‐of‐function mutations in JAZ2 prevent stomatal reopening by COR by inhibiting SA‐dependent stomatal closure triggered upon specific microbial perception without affecting ABA signalling at the stomata (Gimenez‐Ibanez *et al*., 2017). Second, the rate of stomatal aperture in normal light conditions was not affected in *Sljaz2Δjas* tomato mutants. Third, neither water transpiration rate or leaf temperature was affected in *Sljaz2Δjas* compared to WT. Finally, *Sljaz2Δjas* plants displayed a complete phenotypically normal development compared to WT tomatoes (Figure S4). Taken together, these results suggest that the *Sljaz2Δjas* mutation impact on the regulation of stomatal aperture exclusively during biotic conditions, when SA is produced to execute resistance against biotrophic pathogens trying to invade the plant.

Current methods to control bacterial speck disease of tomato are based on removal of plant debris and weeds, crop rotation, and the use of non‐infected seeds and transplants. Chemical sprays based on copper are also commonly used, but these may not be effective in environmental conditions favourable for infection (Somodi *et al*., 1996). Moreover, copper can also kill plant cells if absorbed in sufficient quantities and can produce problematic toxicity in soil. In contrast, tomato resistant varieties offer the most effective means of management. *P. syringae* strains vary in their ability to establish and maintain epiphytic populations on the surface of the leave before infection of internal plant tissues. In this sense, it is becoming clear that transition from epiphytic to endophytic lifestyles is a critical process to establish a successful infection cycle. This transitional process is enhanced by the production of COR. In the case of *Sljaz2Δjas,* these plants would impede the entry on the bacteria, elongating its epiphytic phase where *P. syringae* encounters a harsh environment with limited nutrients. This elongated epiphytic phase on *Sljaz2Δjas* tomatoes should reduce survival of the bacteria further enhancing resistance.

Current resistant varieties are based on the introduction of specific disease‐resistant genes that recognized pathogenic molecules intracellularly. However, pathogens frequently adapt to and overcome genetic resistance especially when it is determined by major resistant genes (Brown, 2015). The ability of *Sljaz2Δjas* tomato plants to interfere with the penetration of the pathogen represents a new strategy for providing resistance that may be achieved by a low energetic cost for the plant and in a clean way for the environment. Ultimately, a hierarchical design containing multiple layers of resistance in a crop may be the most feasible way to achieve durable resistant in the field (Fuchs, 2017; Pilet‐Nayel *et al*., 2017).

Recent results demonstrate that novel techniques for genome editing could be successfully applied in crops to introduce directed resistance. For example, CRISPR/Cas has been recently used to generate powdery mildew resistant tomatoes (Nekrasov *et al*., 2017) and engineer resistance to geminiviruses in tobacco (Ali *et al*., 2015). Public policies are forcing researchers to develop transgene‐free improved crops. In this context DNA editing technologies (also called New Breeding Techniques) have emerged as a novel strategy to overcome this challenge. The status of genetically edited crop varieties is still debated by regulatory authorities, for example countries such as US consider transgene‐free genetically edited crops as non‐GMO. We hope that plant varieties such as the one presented here could be adopted worldwide to enhance plant productivity and resistance against pests. These superior varieties have the potential to fight against agricultural losses in the field due to pests, reducing the chemical inputs towards a more sustainable agriculture for the environment.

Altogether, we demonstrate the feasibility to create bacterial speck resistant tomatoes through CRISPR/Cas9‐mediated editing of *SlJAZ2*. Moreover, we also define a novel strategy to overcome the penetration of COR‐producing *P. syringae* strains through the stomata by spatially uncoupling SA‐JA antagonism.

## Experimental procedures

### Phylogenetic analysis

JAZ proteins sequences from *Arabidopsis thaliana* and *Solanum lycopersicum* (tomato) were aligned with Dialing software (http://www.genomatix.de/cgi-bin/dialign/dialign.pl). Phylogenetic tree was represented using the online tool ‘Phylodendron’, (http://iubio.bio.indiana.edu/treeapp/treeprint-form.html).

### RT‐qPCR analysis

Leaf tissue or epidermal peels were harvested from 4‐ to 6‐week‐old plants and frozen in liquid nitrogen. RNA was extracted using a Plant total RNA purification mini kit (Favorgen^®^). One microgram of RNA was used for cDNA synthesis using a MultiScribe Reverse Transcriptase (Applied Biosystems). PCR was performed on a 7500 thermocycler using SYBR‐green (both from Applied Biosystems). *SlActin* was used as endogenous control. Oligonucleotides used in this study are described in Table S1. Data analysis shown was done using three technical replicates from one biological sample; similar results were obtained with at least two additional independent biological replicates.

### Plasmid construction

Designed gRNAs (Table S2) targeting the Jas domain of *SlJAZ2* (Solyc12 g009220) were cloned into the vector Bsa_Bbs_tandem gRNA_pBS. This vector confers resistance to ampicillin and gRNAs are expressed under different versions of the ubiquitin promoter. Then, a fragment containing both gRNAs and their promoters were cloned into the vector Pk7_CAS9‐TPC_polylinker pBKS. This vector confers resistance to spectinomycin in bacteria and kanamycin in plants. Cas9 is expressed under an ubiquitin promoter. Annealing of gRNAs and cloning was performed by ordinary methods using BsaI and BbsI restriction enzymes. Fragment with the two gRNAs together with their promoters were clone using the KpnI restriction enzyme. gRNAs were designed with the online tool ‘Breaking Cas’ (Oliveros *et al*., 2016) (http://bioinfogp.cnb.csic.es/tools/breakingcas/). gRNAs were unique and specific for the target region (Jas domain sequence of *SlJAZ2*). 1° gRNA had a score of 96.3% with the best possible off‐targets having a 0.2%. 2° gRNA had a score of 98.4%, with the best possible off‐targets having a 0.5%.

### Plant growth and transformation

A tomato cultivar *Moneymaker* was transformed with the pK7_CAS9‐TPC_polylinker pBKS, containing both gRNAs for *SlJAZ2*, as previously described (Wittmann *et al*., 2016). Plants were grown under a 16‐h‐light/8‐h‐dark cycle in a growth chamber or greenhouse. Around 120 cotyledons (60 tomato plants) were initially used for transformation with *Agrobacterium*. Many cotyledons generated a callus and produced shots, which were transferred for root generation. In total, 23 regenerated plants were transferred to the greenhouse. PCR amplification of *SlJAZ2* and DNA sequencing identified mutations in these plants and indicated that 8/23 were wild‐type, 12/23 were heterozygous or chimeras and 3/23 contained an homozygous mutation. T1 and T2 progeny of selected *Sljaz2Δjas* Lines (1 and 2) was further obtained and analysed by DNA sequencing of *SlJAZ2* to confirm that homozygous mutations were stably transmitted to the offspring.

### Measurements of stomatal aperture

After 2 days of stratification of seeds at 4°C, tomato WT and *Sljaz2Δjas* Lines 1 and 2 were grown on soil in a chamber with an 8 h light period at 23°C and a 16 h dark period at 19°C, relative humidity of 75%. The abaxial side of leave from 4‐ to 6‐week‐old tomato plants were collected and stuck on cover slips (peeled). Samples were incubated in a solution of 10 mM MES/Tris pH 6.0 and 30 mM KCl (working buffer). For promotion of closure assays, compounds (10μm flg22, 10μm flg22 + 2μm COR or a mock solution) were directly diluted in the working buffer in contact with epidermal peels during 2 h. Aperture of stomata and hydathode pores were determined under an optical microscope fitted with a camera lucida and a digitizing pad linked to a computer as described (Leonhardt *et al*., 1997). Values reported are the means of at least three independent experiments, for which 60 aperture widths were measured each time. Error bars represent standard deviations of the means. One‐way Anova with Tukey‐HSD was used for statistical analysis. Calculations were performed using a statistical calculator (http://astatsa.com/OneWay_Anova_with_TukeyHSD/).

### Pseudomonas bacterial strains

*Pseudomonas* strains used in this study were *Pseudomonas syringae* pv. *tomato* (*Pto*) DC3000 and the coronatine‐deficient *Pto* DC3000 strain (*Pto* DC3000 *COR−*) which is a *Pto* DC3000 AK87 mutant that carries mutations in cmaA (coronamic acid A) and cfa6 (coronafacic acid 6) (Brooks *et al*., 2004).

### *Pseudomonas syringae* DC3000 infection assays

Tomato plants for infection assays were grown in a cycle of 14 hours light and 10 hours darkness with 45‐60% humidity for 6‐7 weeks. Bacterial growth assays in tomato were performed as described previously with some modifications (Balmuth and Rathjen, 2007). For surface infection assays, plants were dipped in a bacterial suspension containing 10^8^ colony‐forming units m/L (cfu/mL) bacteria (OD_600_ = 0.2) with 0.02% Silwet L‐77. For bacterial growth assays by syringe infiltration, leaves were syringe infiltrated with a bacterial suspension containing 5x10^5^ cfu/mL bacteria (OD_600_ = 0.001). Leaf disks were collected 2 days (infiltration) and 6/7 days (surface infection) postinfection and bacterial growth was quantified as described previously (Gimenez‐Ibanez *et al*., 2009). Error bars indicate SEM (*n* = 7). These experiments were repeated at least three times with similar results, and representative results are shown.

### *Botrytis cinerea* infection assays

*Botrytis cinerea* infection assays were performed as described previously (Monte *et al*., 2014) with some modifications. Five‐ to six‐week‐old tomato leaves were inoculated with 20 μL of a suspension of 5 × 10^6^ spores m/L PDB (Difco, Le Pont de Claix, France). At least eight leaves were inoculated per treatment. Disease symptoms were scored 4 days postinoculations. Area of the lesion was measured using *jimage* software (https://imagej.nih.gov/ij/). This experiment was repeated twice with similar results.

### Leaf temperature and water transpiration ratio

For leaf temperature assays, 3–4 weeks tomato plants were examined under Thermacam infrared camera (FLIR A655sc 25°, 50°Hz) during different days. Images were saved and analysed on a personal computer using the ResearchIR4 Max Software provided by FLIR. For the calculation of water transpiration rate, plants were stratified 2 days at 4°C in dark and grown in soils from 1 to 2 weeks. Then plants were transferred to pots with soil at 20% humidity (all pots weight the same and humidity content was calculated to be 20%) and keep some days for acclimatization. During at least 10 days plants weight was measured every day, after measuring water was added to restore 20% humidity in pots, a control (without plant) was used to measure the amount of water lost by exchange of the soil. The difference of the water lost compare to the negative control (without plant) is a measure of the water transpiration rate of the plants through the stomata. This experiment was repeated twice with similar results.

### Statistical methods

Statistical significance based on Students's *t* test analysis was calculated using Excel software (*P < 0.05; **P < 0.01). Statistical significance based on one‐way Anova with Tukey‐HSD (*P < 0.05; **P < 0.01) was calculated using a statistical calculator (http://astatsa.com/OneWay_Anova_with_TukeyHSD/).

## Conflict of interest

A.O, S.G‐I. and R.S. are inventors in a patent regarding the technology published in this paper.

## Author contributions

R.S. designed the research. A.O, S.G‐I. and N.L. performed the experiments. All authors analyse the data. R.S. supervised the work. A.O., S.G‐I. and R.S. wrote the article. All authors read and edited the article.

## Supporting information


**Figure S1** Phylogenetic tree of all AtJAZs and SlJAZs. The schematic representation indicates length and domains of each JAZ protein.
**Figure S2** False colour infrared image shows no differences between WT and *Sljaz2Δjas* plants. On the left, image of WT and *Sljaz2Δjas* tomato plants (Line 1 and Line 2), as depicted in the figure, taken with a reflex camera. On the right, the same plants analysed under an infrared camera to measure leaf temperature.
**Figure S3** *Sljaz2Δjas* mutant is equally resistant to *Pto* DC3000 COR‐ when surface inoculated. Growth of *Pto* DC3000 COR‐ on WT and *Sljaz2Δjas* (Line 1) tomato plants 7 days after surface inoculation by dipping with bacteria at 10^8^ cfu/mL. Error bars indicate SEM (*n* = 7).
**Figure S4** *Sljaz2Δjaz* plants display a normal phenotype.
**Table S1** RT‐PCR oligonucleotides used in this study.
**Table S2** Oligonucleotides used as gRNAs in this study.
