# Supplementary material for: Design of a bacterial speck resistant tomato by CRISPR/Cas9‐mediated editing of SlJAZ2
Source: Plant Biotechnol J. 2018 Oct 5;17(3):665–73. doi: 10.1111/pbi.13006 (PMC6381780; doi:10.1111/pbi.13006)
Supplement: Supplementary file 1 — Figure S1 Phylogenetic tree of all AtJAZs and SlJAZs. The schematic representation indicates length and domains of each JAZ protein. Figure S2 False colour infrared image shows no differences between WT and Sljaz2Δjas plants. On the left, image of WT and Sljaz2Δjas tomato plants (Line 1 and Line 2), as depicted in the figure, taken with a reflex camera. On the right, the same plants analysed under an infrared camera to measure leaf temperature. Figure S3 Sljaz2Δjas mutant is equally resistant to Pto DC3000 COR‐ when surface inoculated. Growth of Pto DC3000 COR‐ on WT and Sljaz2Δjas (Line 1) tomato plants 7 days after surface inoculation by dipping with bacteria at 108 cfu/mL. Error bars indicate SEM (n = 7). Figure S4 Sljaz2Δjaz plants display a normal phenotype. Table S1 RT‐PCR oligonucleotides used in this study. Table S2 Oligonucleotides used as gRNAs in this study. [file PBI-17-665-s001.pdf]

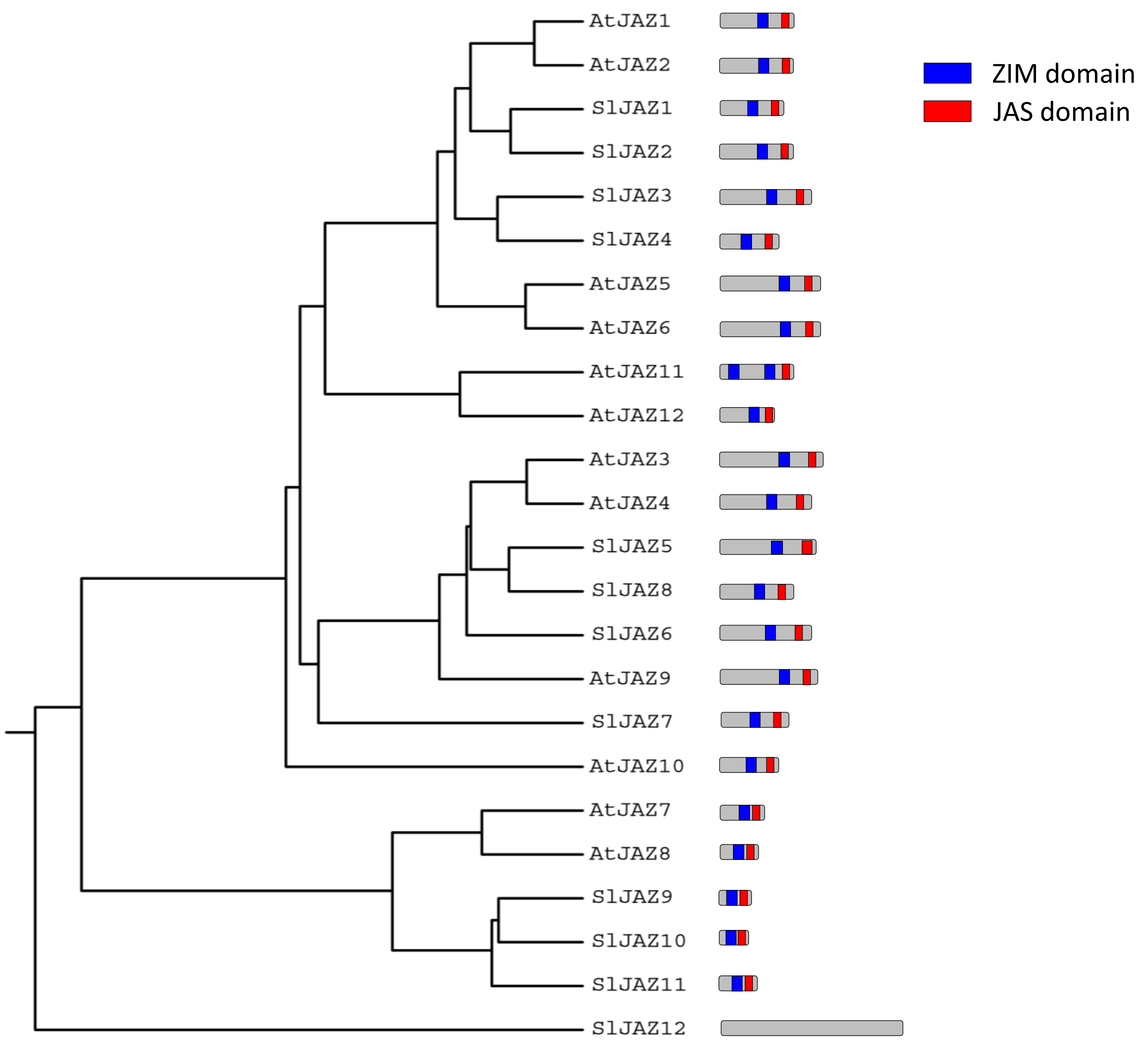

Supplemental Figure 1. Phylogenetic tree of all AtJAZs and SlJAZs. The schematic representation indicates length and domains of each JAZ protein.

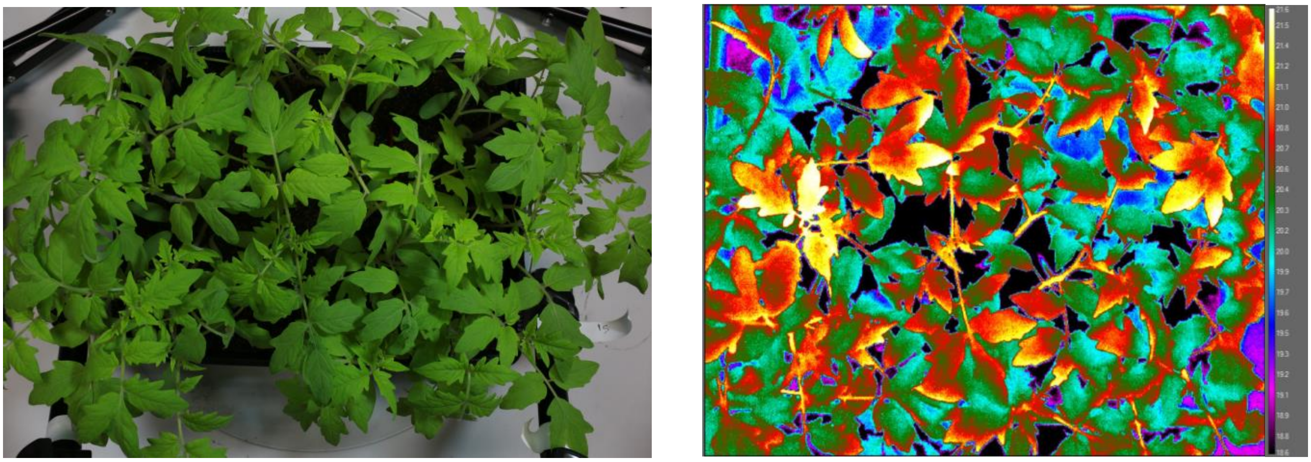

Supplemental Figure 2. False color infrared image shows no differences between WT and *Sljaz2Δjas* plants. On the left, image of WT and *Sljaz2Δjas* tomato plants (Line 1 and Line 2), as depicted in the figure, taken with a reflex camera. On the right, the same plants analyzed under an infrared camera to measure leaf temperature.

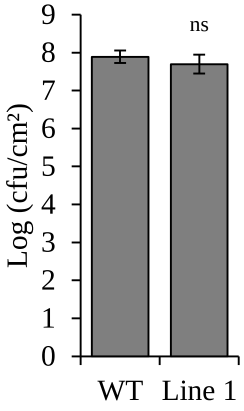

Supplemental Figure 3. ***Sljaz2Δjas*** **mutant is equally resistant to** ***Pto*** **DC3000 COR- when surface inoculated.** Growth of *Pto* DC3000 COR- on WT and *Sljaz2Δjas* (Line 1) tomato plants 7 days after surface inoculation by dipping with bacteria at 10^8 cfu/ml. Error bars indicate SEM (n=7).

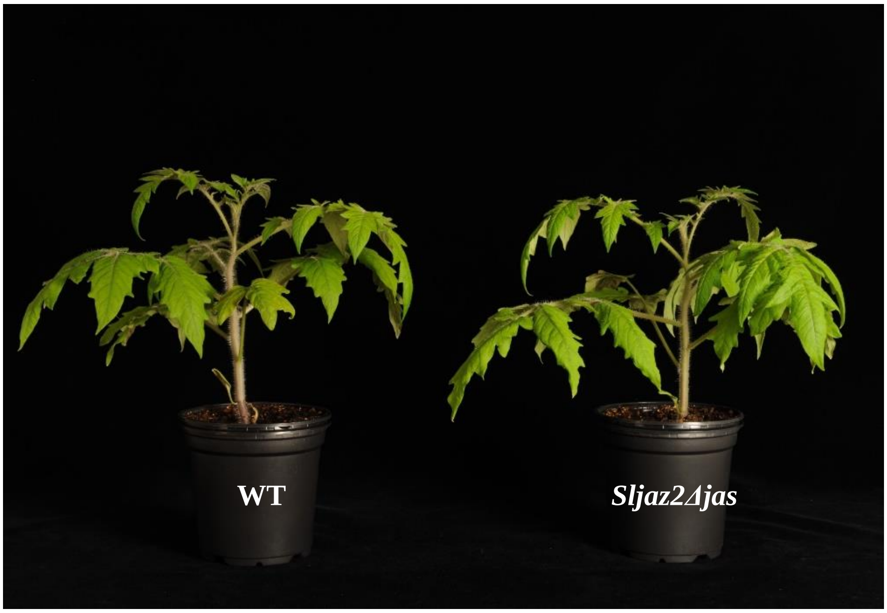

Supplemental Figure 4. *Sljaz2Δjaz* plants display a normal phenotype**.**

**Q-pcr oligonucleotides**	**Sequence**
***SlACTIN* FW**	CAAGTTATTACCATTGGTGCTGAGA
***SlACTIN* RV**	TGCAGCTTCCATACCAATCATG
***SlJAZ1* FW**	GGAAACAATCCTGCTAAACCA
***SlJAZ1* RV**	TCCGAAACTCGGAACCAC
***SlJAZ2* FW**	AAGACAGAATCTTGGAAACCTGA
***SlJAZ2* RV**	AACAATGACTTGTCCACCATAAAA
***SlJAZ3* FW**	AACACCTCCAGATTAAGCCAGAC
***SlJAZ3* RV**	AATTGTGCTTGTGCTGTTGC
***SlJAZ4* FW**	TGGAAAAGCAAATATCAATGATCTAA
***SlJAZ4* RV**	ACAAATCCTTTGTTGCTGAGG
**P1**	CAGCTGATCAATCTGGTGTGA
**P2**	ATCAGCAACAGAAGGCTGTG
**P3**	ATTGGTAAATCAGCAACTGTG
**P4**	GTAAATCAGCAACAGCTGTG

Supplemental Table 1. RT-PCR oligonucleotides used in this study.

Oligonucleotides used as guide RNA	Sequence
**1º gRNA for *SlJAZ2* (A)**	ATTGAAATCAGCAACAGAAGGCTG
**1º gRNA for *SlJAZ2* (B)**	AAACCAGCCTTCTGTTGCTGATTT
**2º gRNA for *SlJAZ2* (A)**	ATTGCTGATTTACCAATCGCGAGA
**2º gRNA for *SlJAZ2* (B)**	AAACTCTCGCGATTGGTAAATCAG

Supplemental Table 2. Oligonucleotides used as gRNAs in this study.
